# Bone Marrow Plasma Cell Assessment before Peripheral Blood Stem Cell Mobilization in Patients with Multiple Myeloma Undergoing Autologous Stem Cell Transplantation

**DOI:** 10.1155/2014/982504

**Published:** 2014-05-06

**Authors:** Sung-Eun Lee, Jae-Ho Yoon, Seung-Hwan Shin, Ki-Seong Eom, Yoo-Jin Kim, Hee-Je Kim, Seok Lee, Seok-Goo Cho, Jong Wook Lee, Woo-Sung Min, Chong-Won Park, Myungshin Kim, Chang-Ki Min

**Affiliations:** ^1^Hematology, Department of Internal Medicine, Seoul St. Mary's Hospital, College of Medicine, The Catholic University of Korea, #222 Banpo-daero, Seocho-Gu, Seoul 137-701, Republic of Korea; ^2^Department of Laboratory Medicine, Seoul St. Mary's Hospital, College of Medicine, The Catholic University of Korea, Seoul 137-701, Republic of Korea

## Abstract

The current definition of complete response (CR) in multiple myeloma (MM) includes negative serum and urine immunofixation (IFE) tests and <5% bone marrow plasma cells (BMPCs). However, many studies of the prognostic impact of pretransplant response have not included BMPCs. We evaluated the prognostic impact of BMPC assessment before peripheral blood stem cell (PBSC) mobilization on subsequent transplant outcomes. BMPCs were assessed by CD138, kappa, and lambda immunostaining in 106 patients. After a median followup of 24.5 months, patients with <5% BMPCs had a significantly better progression-free survival (PFS) compared to those with ≥5% BMPCs (*P* = 0.005). Patients with <5% BMPCs + serologic CR showed superior PFS compared to those with <5% BMPCs + serologic non-CR (*P* = 0.050) or ≥5% BMPCs + serologic non-CR (*P* = 0.001). Interestingly, the prognostic impact of BMPCs was more apparent for patients who did not achieve a serologic CR (*P* = 0.042) compared to those with a serologic CR (*P* = 0.647). We concluded that IFE negativity and <5% BMPCs before PBSC mobilization were important factors to predict PFS in patients with MM undergoing ASCT. Particularly, a significant impact of <5% BMPCs was observed in patients who did not achieve IFE negativity.

## 1. Introduction

The current definition of complete remission (CR) in multiple myeloma (MM) includes negative serum and urine immunofixation (IFE) tests and <5% bone marrow plasma cells (BMPCs) [1]. Additionally, the International Myeloma Working Group (IMWG) has proposed a stringent CR category, requiring a normal serum free light chain (FLC) ratio and the absence of clonal cells in bone marrow [2]. However, there have been several studies aimed at evaluating the role of CR in MM without including BMPCs in the definition of CR [3–5]. Moreover, uncertainty has been introduced by the arbitrary 5% limit in BMPCs in patients with negative IFE.

With the introduction of novel agents for the treatment of MM, the CR rate has increased dramatically and the prognostic impact of CR achievement before autologous stem cell transplantation (ASCT) needs to be evaluated. Kim et al. analyzed 197 MM patients treated with induction chemotherapy followed by a single ASCT and found that CR status before ASCT is an important prognostic factor for better survival outcome. In their study, approach of the CR status did not require a bone marrow examination [5]. However, whether a more precise assessment of CR including bone marrow examination in addition to serologic testing is necessary has not yet been evaluated.

The goal of this study was to determine the prognostic impact of BMPCs as well as serum and urine IFE before peripheral blood stem cell (PBSC) mobilization on the outcomes following ASCT. For this approach, known prognostic factors for survival in MM were also analyzed.

## 2. Materials and Methods

### 2.1. Patient Selection

This study included 106 newly diagnosed MM patients with available bone marrow aspirates and adequate cellularity who underwent ASCT at our institution between June 2009 and September 2012. To assess the prognostic impact of BMPCs as well as serologic response before PBSC mobilization on outcomes following ASCT, patients with MM who had measurable monoclonal (M) protein levels at diagnosis were included in this study and were divided according to achievement of <5% BMPCs and/or negative protein electrophoresis (PEP)/immunofixation (IFE) results in serum and urine. The Institutional Review Board of the Catholic University of Korea approved the research protocol for data analysis and this study was conducted in accordance with the Declaration of Helsinki.

### 2.2. Pretransplant Bone Marrow Examination

**Figure 1:**
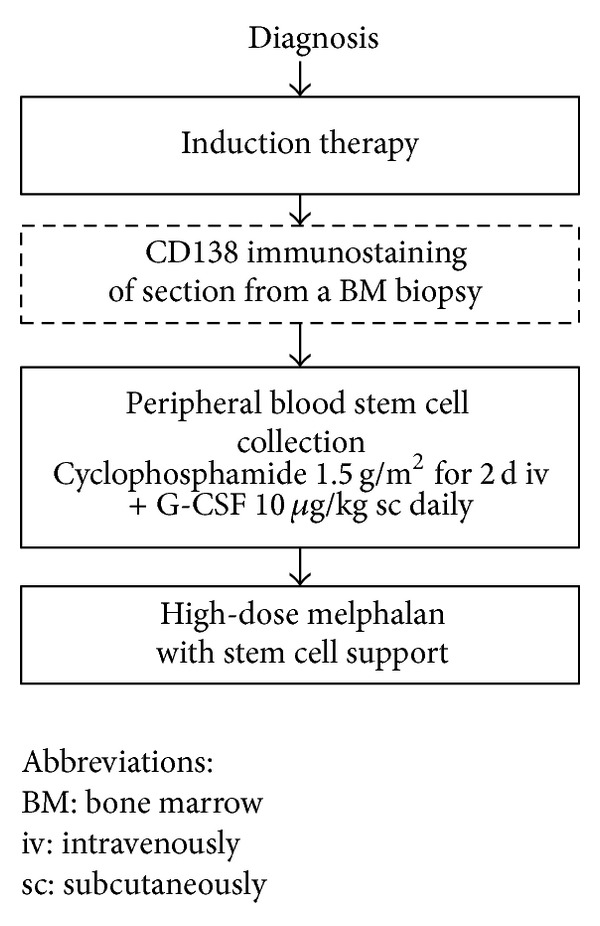
Study scheme.

**Figure 2:**
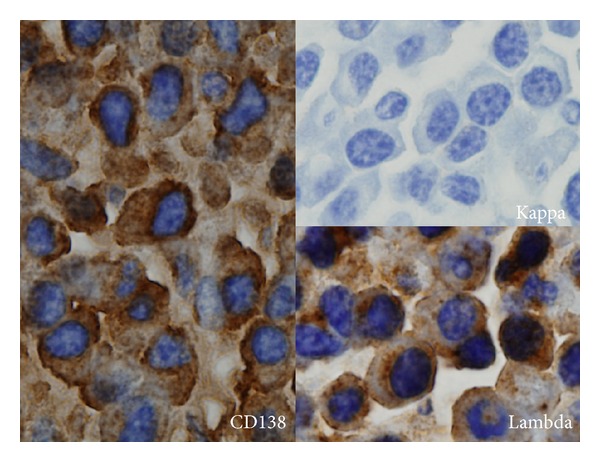
Representative CD138 immunostaining. Plasma cells which express CD138 reveal lambda light chain restriction. CD138, lambda, and kappa chain immunostaining were performed to confirm monoclonal plasma cell on the BM biopsy samples.

BM aspiration and biopsy were performed before PBSC mobilization (Figure 1). Plasma cell counts were assessed after CD138 immunostaining by two examiners blinded to clinical outcomes. Lambda and kappa immunostaining were also performed to confirm monoclonal plasma cell (Figure 2). In all cases of disagreement between examiners, a common reading was organized to achieve a consensus on the count. Differential counts were recorded after a 500-cell count, and marrow cellularity was determined by both BM aspiration and biopsy section.

### 2.3. Treatment Regimens and Transplant Procedures

Induction chemotherapy was consisted of bortezomib ± dexamethasone (*N* = 51), bortezomib + thalidomide + dexamethasone (*N* = 12), thalidomide + dexamethasone (*N* = 31), or high-dose dexamethasone-based regimen (*N* = 12). Transplant timing was dependent on achieving more than a partial response (PR), but some patients without progressive MM resistant to different novel agents (bortezomib and thalidomide) underwent ASCT. General ASCT procedures were performed as described in previous reports [6]. Briefly, all patients were mobilized with cyclophosphamide (3 g/m^2^ total) over 2 days followed by G-CSF (lenograstim, JW pharmaceutical, Seoul, Korea) at 10 *μ*g/kg/day, subcutaneously once a day. Conditioning consisted of melphalan (100 mg/m^2^) for 2 days. G-CSF (5 *μ*g/kg/day) was administered subcutaneously to all patients from one day after transplantation until the absolute neutrophil count (ANC) was >3.0 × 10^9^/L. All patients received prophylactic antibiotics and an antifungal agent (fluconazole) starting 4 days before transplantation until the ANC reached 1.5 × 10^9^/L.

### 2.4. Definitions and Evaluation of Response

Stage was classified by the Durie-Salmon staging system and treatment response was assessed according to the criteria from the International Myeloma Working Group [2]. Serologic CR was defined as a lack of detectable M protein in serum and urine by PEP and IFE. Patients with a deletion of chromosome 13 or hypodiploidy determined by conventional cytogenetic study or t(4;14), t(14;16), and del(17p) established by fluorescent* in situ* hybridization (FISH) of BM samples at diagnosis were stratified as high risk [2]. Overall survival (OS) from transplantation was defined as the time from ASCT to death from any cause, and surviving patients were censored at the last followup. Progression-free survival (PFS) was measured as the time from ASCT to disease progression or death (regardless of cause), whichever came first.

### 2.5. Statistical Analysis

Our main objectives were to evaluate the prognostic impact of BMPCs before PBSC mobilization on the probability of PFS and OS. The probabilities of PFS and OS after ASCT were plotted using the Kaplan-Meier method. Potential prognostic factors for PFS and OS were assessed using a two-tailed log-rank test including age, sex, stage at diagnosis, cytogenetic risk group, serum M protein type, induction therapy, maintenance therapy after ASCT, duration from diagnosis to ASCT, myeloma bone disease apparent on plain radiographs at diagnosis, hemoglobin level, serum LDH, creatinine, calcium, *β*2-microglobulin at diagnosis, and percentage of BMPCs at diagnosis. Covariates having a *P* value of less than 0.1 in the univariate analyses were added to Cox proportional hazards regression models, in which all *P* values were two-sided and statistical significance was set at *P* < 0.05. Associations between categorical variables were assessed using either *χ* ^2^ or Fisher's exact test. The Mann-Whitney *U* test was used to compare continuous variables.

## 3. Results

### 3.1. Patient Characteristics

A total of 106 MM patients were included in this study, of whom 59 (56%) were male and 47 (44%) were female. The median age was 56 years (range 33–65 years) and the median disease duration before ASCT was 6.7 months (range 2.9–15.6 months). Stage IIA, IIIA, and IIIB diseases at diagnosis comprised 6%, 77%, and 17% of subjects, respectively, and all patients had measurable disease at diagnosis. Among 106 patients, 39 patients (37%) had a negative IFE test in serum and urine before PBSC mobilization, while 38 (36%) and 25 (23%) of patients achieved a very good partial response (VGPR) and PR, respectively. Four patients with MM resistant to different novel agents were in the stable disease (SD).  Table 1 lists the demographic information for all patients and subgroups according to BMPCs before PBSC mobilization. BM examination performed a median of 2.0 months before ASCT (range 0.4–3.7 months). Seventy-three patients (69%) had BMPCs < 5% and 33 patients (31%) showed BMPCs ≥ 5%. The characteristics between the two groups differed in administered type of induction therapy and serologic response before PBSC mobilization (Table 1).

### 3.2. Relation between Serologic CR and BMPCs before ASCT

**Figure 3:**
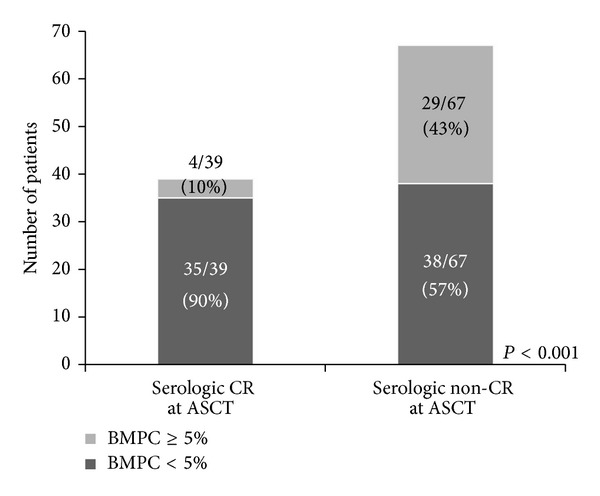
Relation between serologic CR and BMPCs before PBSC mobilization.

Figure 3 shows the relation between serologic CR and BMPCs before PBSC mobilization. The median percentage of BMPCs was 1% (range 0–34%) and 4% (range 0–92%) for patients in serologic CR and non-CR, respectively (*P* < 0.001). Among 39 patients with serologic CR, 35 (90%) had <5% BMPCs, whereas four (10%) had ≥5% BMPCs. The serologic non-CR consisted of <5% BMPCs (*N* = 38, 57%) and ≥5% BMPCs (*N* = 29, 43%; *P* < 0.001). In addition, among 62 patients who had normal serum FLC ratio, 14 (23%) and 48 (77%) patients had ≥5% BMPCs and <5% BMPCs, respectively. For the 44 patients with abnormal serum FLC ratio, 19 (43%) and 25 (57%) patients had ≥5% BMPCs and <5% BMPCs, respectively.

### 3.3. Transplant Outcomes and Prognostic Factors

**Figure 4:**
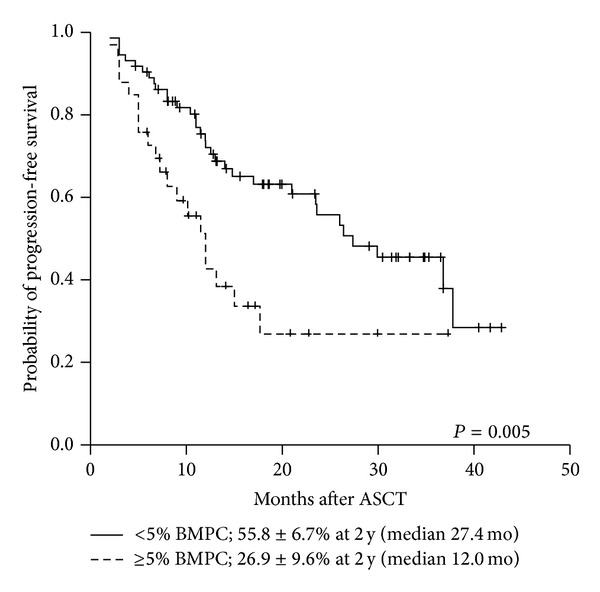
Progression-free survival according to premobilization bone marrow plasma cells.

The median followup for survivors was 24.5 months (range 4.7–45.9 months). All patients achieved engraftment and no patients died of complications before engraftment. The 2-year PFS of patients with <5% BMPCs and ≥5% BMPCs was 55.8% and 26.9% (median PFS 27.4 versus 12.0 months; *P* = 0.005), respectively (Figure 4). The 2-year OS of patients with ≥5% BMPCs and <5% BMPCs were 89.2% and 84.0% (median OS was not reached; *P* = 0.622), respectively.

To assess prognostic factors affecting PFS and OS, univariate and multivariate analyses were performed. High risk cytogenetics (*P* = 0.011), non-CR serologic response before PBSC mobilization (*P* = 0.005), and ≥5% BMPCs before PBSC mobilization (*P* = 0.006) were potential risk factors for a lower PFS (Table 2). Considering the relationships between serologic response and BMPC percentage, patients were reclassified as four groups (<5% BMPCs + serologic CR versus <5% BMPCs + serologic non-CR versus ≥5% BMPCs + serologic CR versus ≥5% BMPCs + serologic non-CR) and entered into the multivariate models. Among the factors that affected PFS determined by univariate analyses, multivariate analyses revealed that high risk cytogenetics (RR of 2.05, *P* = 0.024) was a predictive factor for a lower PFS. Based on the association of BMPCs with serologic response, <5% BMPCs + serologic non-CR (RR of 2.02, *P* = 0.050) and ≥5% BMPCs + serologic non-CR (RR of 3.60, *P* = 0.001) were significantly associated with inferior PFS, compared to <5% BMPCs + serologic CR (Table 3). In addition of the factors affecting OS in the univariate analyses, high risk cytogenetics (RR of 3.92, *P* = 0.024) was an independent factor for OS.

### 3.4. Separate Analysis according to Serologic Subgroups

**Figure 5:**
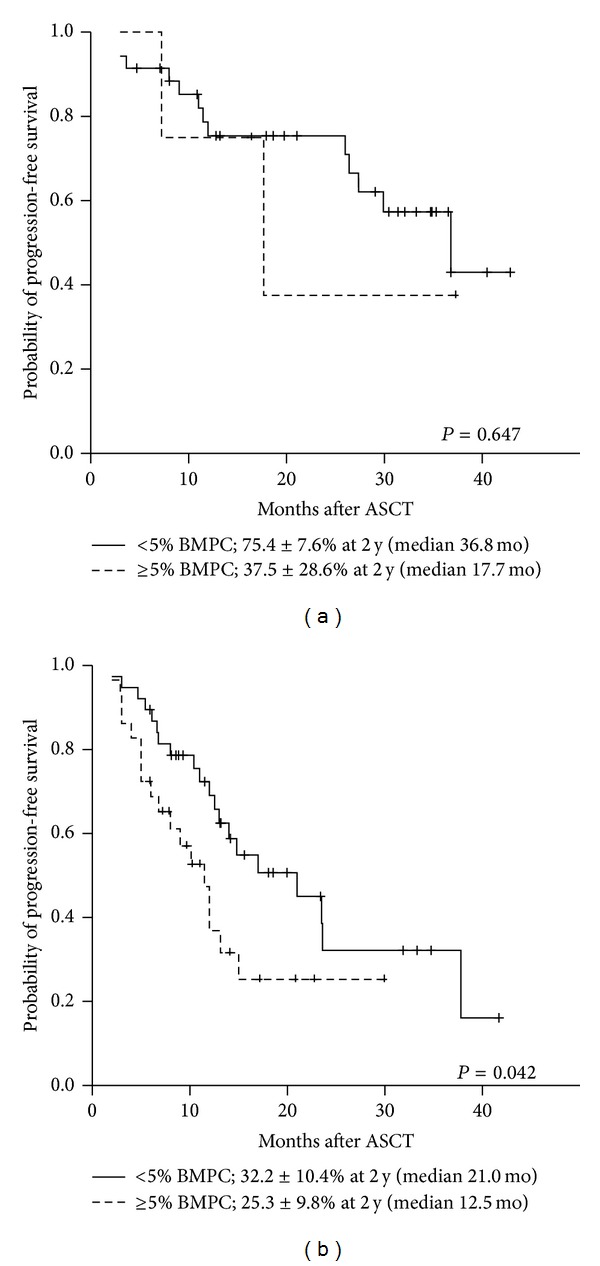
Progression-free survival according to premobilization bone marrow plasma cells in serologic subgroups. (a) Progression-free survival in the serologic CR group and (b) Progression-free survival in the serologic non-CR group.

To minutely evaluate the importance of premobilization BMPCs in assessing the prognosis after ASCT, we performed a separate analysis in two subgroups according to serologic response before PBSC mobilization (serologic CR group versus serologic non-CR group). In the serologic CR subgroup, the 2-year PFS rates were 75.4% and 37.5% (median PFS 36.8 versus 17.7 months; *P* = 0.647) for patients with <5% BMPCs and ≥5% BMPCs, respectively (Figure 5(a)). On the other hand, in the serologic non-CR subgroup, the 2-year PFS rates were 32.2% and 25.3% (median PFS 21.0 versus 12.5 months; *P* = 0.042) for patients with <5% BMPCs and ≥5% BMPCs, respectively (Figure 5(b)).

## 4. Discussion

Over the past 10–15 years, novel agents including immunomodulating drugs and proteasome inhibitors have been increasingly incorporated during ASCT for MM. The existing criteria for the assessment of disease response have not proven entirely satisfactory for the analysis of disease outcome before and after ASCT. CR has hitherto been defined as absence of detectable paraprotein in serum and urine according to IFE, maintained for a minimum of 6 weeks, together with <5% BMPCs on the basis of a normal number of plasma cells in the bone marrow (i.e., <4–5%) [1, 7]. However, the arbitrary 5% limit in BMPCs in patients with negative IFE results can lead to uncertainty. Moreover, the impact of BMPCs on patients with positive IFE results has remained unclear in the context of ASCT for MM. This study demonstrated that in addition to PEP and IFE in serum and urine, a pretransplant conventional BM study with CD138 immunostaining constitutes a predictor for disease progression in patients with MM undergoing ASCT. The prognostic impact of BMPCs was more apparent for patients who did not achieve a serologic CR compared to those with a serologic CR.

In this study, a serologic CR was defined as negative IFE for both serum and urine after induction therapy without considering BMPCs. Patients who had no detectable paraprotein on PEP without a negative IFE result (IFE either positive or not performed) were no longer classified as CR. Although it would be very unusual to achieve a serologic CR in secretory myeloma patients with persisting marrow plasma cell infiltration, we were able to detect this possibility by a BM examination in a small number of patients (*n* = 4, 3.8%). CD138 immunostaining was used to objectively evaluate BMPCs because normal plasma cell morphology is not specified and morphological assessment was thought to be too subjective. Additionally, lambda and kappa chain stains were performed to confirm monoclonal plasma cell. CD138/syndecan-1 is a cell membrane proteoglycan that functions as a matrix receptor and is expressed on the surface of mature epithelial cells [8] as well as normal and neoplastic plasma cells [9]. Within the spectrum of hematologic disorders, CD138 expression is highly sensitive and specific for plasmacytic differentiation and represents an excellent marker for routine evaluation of tissue samples for plasma cell disorders. CD138 is particularly helpful for plasma cell quantification in bone marrow biopsy specimens [10]. CD138 expression, however, has been regarded as heterogeneous and nonspecific for tumor type. Therefore, caution is required in the interpretation of CD138^+^ neoplasms for which a hematolymphoid derivation has not been established, particularly because some nonhematopoietic neoplasms might exhibit plasmacytoid features.

The use of autologous PBSCs for transplantation is associated with the risk of contamination of the graft with tumor cells; whether this impacts response rates, PFS, and OS is still debatable [11]. In this study, we could not determine whether high contamination of grafts with malignant cells [12] or* in vivo* tumor mass prior to ASCT [13] contributed to an unfavorable disease course after transplantation. In particular, the association of the percentage of pretransplant BMPCs with posttransplant PFS in patients having M protein after induction therapy has been unclear. Here, the percentage of BMPCs determined before PBSC mobilization for ASCT by means of CD138 immunostaining was associated with PFS, especially in patients who did not achieve serologic CR (Figure 5(b)). In our study, 38 of 106 patients (35.9%) had a serologic non-CR with a fraction of plasma cells <5% in BM and their survival was better than 29 patients (27.4%) with a serologic non-CR + ≥5% BMPCs. This result reflects the fact that the counting method of BMPCs using immunochemical staining of CD138, kappa, and lambda chain might detect the monoclonal plasma cells producing M protein more sensitively than usual stain such as Wright-Giemsa stain. The possible explanation was that measurement of the M-component reflects the secreted product. Our data suggested that BMPCs should be measured in patients who did not achieve a negative IFE result before PBSC collection. Additional treatment would then be required for those patients with high BMPCs.* In vivo* tumor cell purging prior to mobilization chemotherapy might be one strategy to improve the time to progression of high risk patients. Several studies have been performed to detect minimal residual disease to identify and predict patients at risk for future relapse [14, 15].

A meta-analysis found a strong association between maximal response to induction therapy and long-term survival [16]. To our knowledge, sensitivity to induction therapy measured by BMPCs at the time of ASCT has not been appropriately evaluated. Serial BM examinations are helpful, although the patchy nature of marrow involvement in myeloma makes it difficult to accurately interpret small changes in the percentage of plasma cells present. In this study, the prognostic impact of BMPC determination after induction therapy was more apparent for patients who did not achieve serologic CR compared to those with serologic CR, suggesting that the evaluation of BMPCs after induction therapy is required for all patients to predict disease progression after ASCT as well as to confirm the achievement of CR.

## 5. Conclusions

IFE negativity and <5% BMPCs prior to PBSC mobilization are important factors to predict PFS in patients with MM undergoing ASCT. A premobilization conventional BM study with CD138 immunostaining may constitute a useful predictor for disease progression in patients who did not achieve IFE negativity.

## Figures and Tables

**Table 1:** Characteristics of patients and treatment.

	All patients (*N* = 106) (%)	BMPCs before PBSC mobilization		*P* value
		<5% (*N* = 73)	≥5% (*N* = 33)	
Baseline characteristics				
Age, years, median (range)	56 (33–65)	56 (34–65)	56 (33–65)	ns
Patient gender (M/F)	59 (56)/47 (44)	39 (53)/34 (47)	20 (61)/13 (39)	ns
Serum M protein				ns
Light chain	30 (28)	20 (28)	10 (30)	
Other than light chain	76 (72)	53 (72)	23 (70)	
Stage at diagnosis				ns
IIA	6 (6)	6 (8)	0 (0)	
IIIA/B	100 (94)	67 (92)	33 (100)	
Cytogenetics*				ns
Standard risk	88 (83)	63 (86)	25 (76)	
High risk	18 (17)	10 (14)	8 (24)	
BMPCs at diagnosis, %, median (range)	39 (3–90)	38 (3–88)	42.3 (8–90)	ns
Myeloma bone disease on plain radiographs, yes/no	81 (76)/25 (24)	58 (80)/15 (20)	23 (70)/10 (30)	ns
Serum calcium at diagnosis, mg/dL, median (range)	8.9 (6.8–16.2)	8.9 (6.8–15.7)	9.0 (7.4–16.2)	ns
Serum creatinine at diagnosis, mg/dL, median (range)	0.9 (0.4–8.3)	0.9 (0.4–8.3)	0.9 (0.5–5.6)	ns
*β*2-microglobulin at diagnosis, mg/mL, median (range)	3.5 (0.2–41.4)	3.5 (0.2–41.4)	3.8 (1.6–36.0)	ns
Hemoglobin at diagnosis, g/dL, median (range)	9.8 (6.0–17.2)	9.8 (6.0–17.2)	9.5 (6.5–14.8)	ns
Serum lactate dehydrogenase at diagnosis, U/L, median (range)	374 (81–1054)	389 (165–1054)	361 (81–889)	ns

Treatment and outcomes				
Induction therapy				0.039
Bortezomib ± dexamethasone	51 (48)	36 (49)	15 (46)	
Bortezomib + thalidomide + dexamethasone	12 (11.5)	8 (11)	4 (12)	
Thalidomide + dexamethasone	31 (29)	17 (23)	14 (42)	
High-dose dexa-based regimen	12 (11.5)	12 (17)	0 (0)	
Duration from diagnosis to ASCT, months, median (range)	6.7 (2.9–15.6)	6.9 (2.9–15.6)	6.1 (3.9–15.3)	ns
Maintenance therapy after ASCT				0.742
Yes	73 (69)	51 (70)	22 (67)	
Thalidomide	50 (47)	35 (48)	15 (46)	
Bortezomib or Lenalidomide	8 (8)	3 (4)	5 (15)	
Prednisolone	15 (14)	13 (18)	2 (6)	
No	33 (31)	22 (30)	11 (33)	
Serologic response before PBSC mobilization				<0.001
CR	39 (37)	35 (48)	4 (12)	
VGPR	38 (36)	25 (34)	13 (39.5)	
PR	25 (23)	12 (17)	13 (39.5)	
SD	4 (4)	1 (1)	3 (9)	
Serologic response after PBSC mobilization				<0.001
CR	47 (44)	41 (56)	6 (18)	
VGPR	36 (34)	22 (30)	14 (42)	
PR	20 (19)	10 (14)	10 (30)	
SD	3 (3)	0 (0)	3 (9)	

ASCT: autologous stem cell transplantation; BMPCs: bone marrow plasma cells; CR: complete response; ns: not significant; PBSC: peripheral blood stem cell; PR: partial response; SD: stable disease; VGPR: very good partial response.

*High risk cytogenetics is defined as hypodiploidy or deletion 13 on conventional cytogenetics or presence of t(4; 14), t(14; 16), and del(17p) on fluorescent *in  situ* hybridization and/or conventional cytogenetics. All other cytogenetic abnormalities were considered standard risk.

**Table 2:** Univariate analysis of potential factors affecting survival outcomes.

Potential factor	Progression-free survival		Overall survival	
	RR (95% CI)	*P* value	RR (95% CI)	*P* value
Age at diagnosis (<60 years versus ≥60 years)	0.86 (0.46–1.58)	0.624	0.55 (0.12–2.51)	0.439
Sex (F versus M)	1.09 (0.63–1.90)	0.750	4.41 (0.96–10.13)	0.056
Immunoglobulin type (others versus Light chain only)	1.01 (0.56–1.82)	0.965	1.15 (0.35–3.83)	0.816
Durie-Salmon stage at diagnosis (II versus III)	1.08 (0.57–2.06)	0.821	1.19 (0.32–4.43)	0.791
Cytogenetics (standard versus High risk)	2.22 (1.20–4.10)	0.011	5.35 (1.71–16.71)	0.004
BMPCs at diagnosis (%) (continuous)	1.00 (0.99–1.01)	0.549	0.99 (0.96–1.01)	0.254
Serum calcium at diagnosis (<10 mg/dL versus ≥10 mg/dL)	1.59 (0.85–2.97)	0.151	3.42 (1.09–10.78)	0.036
Serum creatinine at diagnosis (<2 mg/dL versus ≥2 mg/dL)	0.81 (0.38–1.72)	0.583	1.73 (0.47–6.40)	0.413
*β*2-microglobulin at diagnosis (<5.5 mg/dL versus ≥5.5 mg/dL)	1.56 (0.89–2.71)	0.119	1.36 (0.43–4.30)	0.601
Hemoglobin at diagnosis (≥10 g/dL versus <10 g/dL)	1.20 (0.69–2.08)	0.524	1.17 (0.37–3.70)	0.786
Serum lactate dehydrogenase at diagnosis (<450 U/L versus ≥450 U/L)	1.47 (0.85–2.56)	0.169	1.58 (0.50–5.00)	0.433
Induction therapy (novel agents* versus nonnovel)	0.97 (0.45–2.06)	0.927	0.50 (0.06–3.86)	0.504
Duration from diagnosis to ASCT (<6 mo versus ≥6 mo)	0.79 (0.45–1.39)	0.412	0.65 (0.21–2.05)	0.460
Maintenance therapy after ASCT (no versus yes)	1.02 (0.54–1.93)	0.945	0.79 (0.21–2.97)	0.723
Serologic response before PBSC mobilization (CR versus non-CR)	2.41 (1.30–4.45)	0.005	2.27 (0.61–8.44)	0.222
Serologic response after PBSC mobilization (CR versus non-CR)^†^	2.77 (1.53–5.04)	0.001	1.96 (0.59–6.55)	0.274
BMPCs before PBSC mobilization (<5% versus ≥5%)	2.23 (1.26–3.95)	0.006	1.35 (0.40–4.53)	0.623
Response before PBSC mobilization^‡^ (<5% + CR versus <5% + non-CR versus ≥5% + CR versus ≥5% + non-CR)	1.98 (0.97–4.02) 1.44 (0.32–6.41) 3.83 (1.81–8.11)	0.005	3.45 (0.69–17.18) 4.94 (0.44–54.99) 2.58 (0.43–15.61)	0.443

ASCT: autologous stem cell transplantation; BMPCs: bone marrow plasma cells; CI: confidential interval; CR: complete response; non-CR: noncomplete response; RR: relative risk.

*Novel agents for induction therapies included proteasome inhibitors and immunomodulatory drug.

^†^Serologic responses before and after PBSC mobilization were correlated. Therefore, serologic response after PBSC mobilization was not entered into the multivariate model.

^‡^BMPCs + serologic CR.

**Table 3:** Multivariate analysis of independent factors affecting survival outcomes.

Variable	E/N*	RR (95% CI)	*P* value
Progression-free survival			
Cytogenetics			
Standard	39/88	1	
High risk	14/18	2.05 (1.10–3.84)	0.024
Response before PBSC mobilization†			
<5% + CR	13/35	1	
<5% + non-CR	20/38	2.02 (1.00–4.11)	0.050
≥5% + CR	2/4	1.52 (0.34–6.78)	0.583
≥5% + non-CR	18/29	3.60 (1.69–7.63)	0.001
Overall survival			
Sex			
Male	10/59	1	
Female	2/47	0.29 (0.06–1.33)	0.109
Cytogenetics			
Standard	6/88	1	
High risk	6/18	3.92 (1.20–12.81)	0.024
Ca at diagnosis			
<10 mg/dL	7/87	1	
≥10 mg/dL	5/19	2.14 (0.65–7.06)	0.214

CR: complete response; non-CR: noncomplete response; RR: relative risk.

*E/N: number of events/number of evaluable patients.

^†^BMPCs + serologic CR.
